# Use of Enzymatic Bio-Fenton as a New Approach in Decolorization of Malachite Green

**DOI:** 10.1100/2012/691569

**Published:** 2012-05-02

**Authors:** Afzal Karimi, Mostafa Aghbolaghy, Alireza Khataee, Shabnam Shoa Bargh

**Affiliations:** ^1^Department of Chemical Engineering, University of Tabriz, Tabriz 51666-17615, Iran; ^2^Department of Applied Chemistry, University of Tabriz, Tabriz 51666-17615, Iran

## Abstract

An enzymatic reaction using glucose oxidase was applied for *in situ* production of hydrogen peroxide for use in simultaneously Fenton's reaction in decolorization of malachite green. It was found that decolorization rate increased by increasing of glucose concentration from 0.2 g/L to 1.5 g/L. Decolorization rate showed different behaviors versus temperature changes. Initial rate of decolorization process was increased by increasing of temperature; after 30 minutes, especially at temperatures above 30°C, the decolorization rate was gradually reduced. The pH value in the reaction media was decreased from natural to about pH = 3 which had synergic effect on the Fenton process by stabilizing of Fe^2+^ ions.

## 1. Introduction

Releasing of chemical wastes into the environment has caused a variety of environmental problems. Some of the important industrial pollutants are dyes. Dyestuffs are vastly used in the textile, cosmetics, paper, leather, plastics, pharmaceutical, and food industries [1, 2]. The textile industry accounts approximately two-thirds of total dyestuff market [3], and it is estimated that as much as 2–50% of applied dyestuff may be lost to wastewater which is finally released into environment [4, 5]. Dyeing materials are major sources of environmental contamination, especially for water pollution [6]. Therefore, the removal of dyestuffs from waste effluents becomes environmentally important. However, there is no simple and economical method for color removal from textile effluents. Many methods such as ozonation, photooxidation, electrocoagulation, adsorption, activated carbon, froth flotation, reverse osmosis, ion exchange, membrane filtration, and flocculation have been tested for removing of color from textile effluents [7–9]. Nevertheless, expensive plant requirements, high operational costs, regeneration problem, secondary pollutants, sensitivity to variations in wastewater input, interference by other wastewater constituents, and residual sludge generation are some technological and economical disadvantages of these methods [10–14].

Malachite green (MG) belongs to triphenyl methane dyes and is the most applicable material in dyeing industry [15]. It is known that MG has damaging effects on ecosystem and its releasing into aqueous streams will affect aquatic life and cause impairing effects in liver, kidney, intestine, and gonads [10, 16]. In addition, MG is tumor promoter to human beings and contact of MG with skin causes irritation, redness, and pain [17, 18]. Therefore, removing of MG from contaminated effluents is necessary for ecosystem and human health.

Although conventional decolorization processes are not suitable enough, biological methods are attractive alternatives which have received increasing interest owing to their cost, effectiveness, selectivity, capability of complete degradation of organic pollutants, and *in situ *use [4, 20–22]. On the other hand, Fenton's reaction is a well-known and powerful method for oxidation of organic pollutants [23]. In this reaction, ferrous ion reacts with hydrogen peroxide to produce hydroxyl radical through the following equation [24]:

(1) $${\text{Fe}}^{2+}+{\text{H}}_{2}{\text{O}}_{2}\to {\text{Fe}}^{3+}+{\text{HO}}^{∙}+{\text{HO}}^{-}.$$

Hydroxyl radicals have powerful oxidizing potential (E° = 2.8 V), so they can easily oxidize a wide diversity of organic materials [25]. As have been reported in literatures, Fenton's reaction has been used to treat various wastewaters containing dyestuffs [26–28].

In this study, the feasibility of bio-Fenton reaction, which is a novel approach in decolorization of MG, was investigated. A new simple enzymatic reaction, catalyzed by glucose oxidase (GO_X_), was applied for *in situ *production of hydrogen peroxide and simultaneously Fenton's reaction performed for decolorization of the dye. The effects of various factors including substrate concentration, initial dye concentration, and temperature on the efficiency of bio-Fenton reaction in the removing of MG from aqueous solution were investigated. In addition, variation of pH in bio-Fenton process and its effect on MG decolorization were monitored.

## 2. Materials and Methods

### 2.1. Materials

**Figure 1:**
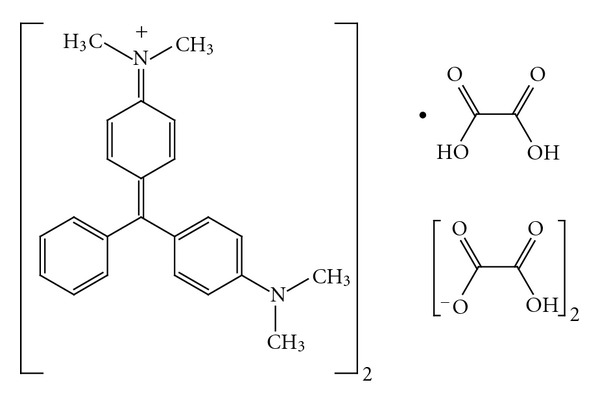
chemical structure of malachite green [19].

Glucose oxidase (GO_X_) type II (EC 1.1.3.4, 25 U/mg, from *Aspergillus niger*), *β*-D-(+)-glucose, 2,2′-Azino-di-[3-ethylbenzthiazoline sulfonate], FeSO_4_, and malachite green oxalate (MG) were obtained from Sigma Aldrich. The chemical structure of the dye has been represented in Figure 1. All chemicals were of analytical grade, and deionized water was used in preparation of solutions.

### 2.2. Dye Concentration Analysis

**Figure 2:**
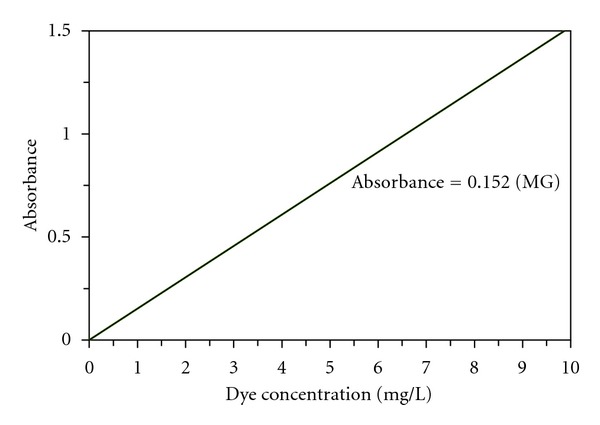
Calibration curve.

The concentration of MG in sample solutions was determined by measuring its absorbance with spectrophotometer (UV/Vis spectrophotometer WPA light wave S2000) at maximum absorption wavelengths of the dye (*λ*_max⁡_ = 619 nm). Different standard solutions were used to plot absorbance calibration curve, which is shown in Figure 2.

**Figure 3:**
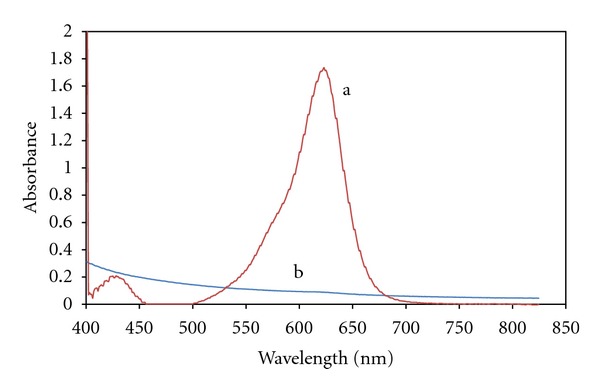
Absorbance spectrum: (a); aquatic solution of MG (b); aquatic solution of glucose, glucose oxidase and Fe^2+^.

As has been shown in Figure 3, the absorbance spectrum of reaction solution without MG is different enough from absorbance pick of aquatic solution of MG, so concentration of MG can be measured accurately.

The decolorization efficiency was calculated through

(2) $$R\left(\%\right)\;=\left(\frac{C_{0}-C_{t}}{C_{0}}\right)\times 100,$$

where *C*_0_ is the initial concentration of MG (mg/L) and *C* is the concentration of MG (mg/L) at time *t* (min).

The decolorization rate was also calculated by (3).

(3) $$r=\left(\frac{{\left(\text{color}\;\text{removal}\;\%\right)}_{t_{2}}-\;{\left(\text{color}\;\text{removal}\;\%\right)}_{t_{1}}}{t_{2}-t_{1}}\right)*C_{0}.$$

### 2.3. Assay of GO_X_ Activity

In the presence of oxygen, GO_X_ oxidizes *β*-D-glucose to *β*-D-glucono-*δ*-lactone and H_2_O_2_. The produced H_2_O_2_ is then utilized to oxidize a chromogenic substrate in the presence of catalase. 2,2′-Azino-di-[3-ethylbenzthiazoline sulfonate] was used for monitoring color change through forming a greenish-blue oxidized product that was measured spectrophotometrically at 420 nm. One unit of catalyst activity (U) is defined as the amount of GO_X_ required to consume 1 *μ*mol substrate in one min at 25°C [29].

### 2.4. Experimental Procedures

Experiments were performed in 250 mL Erlenmeyer flasks containing 50 mL of reaction mixtures at constant shaking rate of 160 r/min. Reaction mixtures were prepared by desired amounts of MG, glucose oxidase, glucose, and FeSO_4_ in distilled water. The effects of glucose concentration (0.2–1.5 g/L), MG concentration (5–40 mg/L) and temperature (15–40°C), on the efficiency of bio-Fenton decolorization process were studied without any control on pH value.

## 3. Results and Discussion

### 3.1. Bio-Fenton Process

In this process, glucose oxidase in presence of glucose was applied for *in situ* production of hydrogen peroxide through (4) and simultaneously hydroxyl radicals producing from Fenton's reaction were used for decolorization of MG through

(4) $$\begin{array}{c} {\text{C}}_{6}{\text{H}}_{12}{\text{O}}_{6}+\;{\text{H}}_{2}\text{O}+\;{\text{O}}_{2}\overset{{\text{GO}}_{\text{X}}}{\to }{\text{C}}_{6}{\text{H}}_{12}{\text{O}}_{7}+{\text{H}}_{2}{\text{O}}_{2} \end{array}$$

(5) $$\begin{array}{c} {\text{Fe}}^{2+}+{\text{H}}_{2}{\text{O}}_{2}\to {\text{Fe}}^{3+}+{\text{HO}}^{∙}+{\text{HO}}^{-} \end{array}.$$

### 3.2. Effect of Glucose Concentration

**Figure 4:**
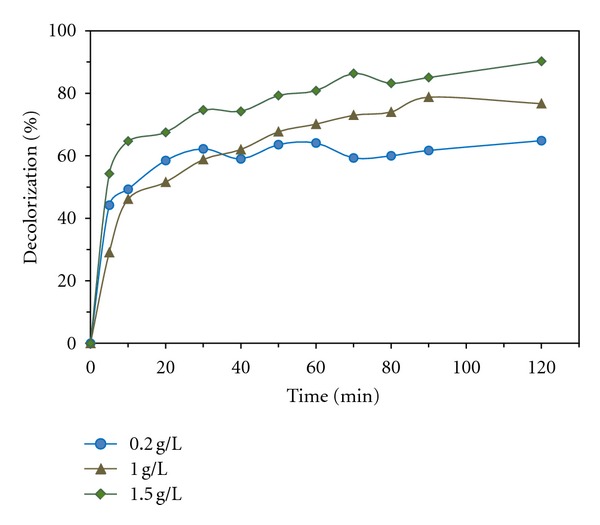
Decolorization percentage at various concentrations of glucose at [MG]_0_=15 mg/L, [enzyme]_0_=1000 U/L, [Fe^2+^]_0_ = 10 mM, and T = 30°C.

As can be seen in (4), production of hydrogen peroxide is dependent on glucose concentration. For study of the effect of glucose concentration on decolorization rate, different concentrations of glucose (0.2, 1, and 1.5 g/L) were tested. This experiments was conducted at initial MG concentration of 15 mg/L, 1000 U/L enzyme, and 10 mM [Fe^2+^]_0_ at 30°C. As can be seen in Figure 4, increasing in glucose concentration has caused increasing in decolorization rate. Because of gradual production and immediate consumption of hydrogen peroxide in bio-Fenton process, undesired reactions such as scavenging effect of H_2_O_2_ (5)–(8) could not be conducted at low or medium concentrations of H_2_O_2_ [30]

(6) $$\begin{array}{c} {\text{H}}_{2}{\text{O}}_{2}+\;{\text{OH}}^{∙}\to {{\text{HO}}_{2}}^{∙}+{\text{H}}_{2}\text{O} \end{array}$$

(7) $$\begin{array}{c} {\text{H}}_{2}{\text{O}}_{2}+\;{{\text{OH}}_{2}}^{∙}\to {\text{OH}}^{∙}+{\text{O}}_{2}+{\text{H}}_{2}\text{O} \end{array}$$

(8) $$\begin{array}{c} {\text{OH}}^{∙}+\;{{\text{OH}}_{2}}^{∙}\to {\text{O}}_{2}+{\text{H}}_{2}\text{O} \end{array}.$$

### 3.3. Effect of Initial MG Concentration

**Figure 5:**
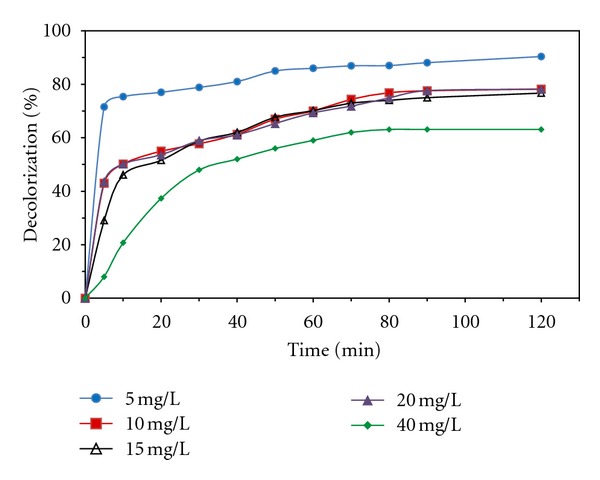
Decolorization percentage at various concentrations of MG at [Glucose]_0_=1 g/L, [enzyme]_0_=1000 U/L, [Fe^2+^]_0_ = 10 mM, and T = 30°C.

Effect of initial MG concentration on decolorization process was tested using different initial concentrations of MG (5, 10, 15, 20, and 40 mg/L). This experiment was conducted at glucose concentration of 1 g/L, 1000 U/L enzyme, and 10 mM [Fe^2+^]_0_ at 30°C. The results have been shown in Figure 5. As can be seen, by increasing the initial MG concentration, decolorization efficacy decreased. But in terms of decolorization rates, which have been reported in Table 1, it is observed that decolorization rate at various periods of time was maximum for initial MG concentration of 40 mg/L and was minimum for 5 mg/L. It is mainly because there are high numbers of dye molecules in high concentration of MG while other conditions are constant. The other reason is the fast consumption of hydroxyl radicals in high concentrations of the dye, so probability of undesired reactions of radicals is low.

### 3.4. Effect of Temperature

The effect of temperature on decolorization process was performed at 15, 30, 35 and 40°C This experiment was conducted at initial MG concentration of 15 mg/L, glucose concentration of 1 g/L, 1000 U/L enzyme, and 10 mM [Fe^2+^]_0_. The results have been shown in Table 2. In higher temperatures, initial decolorization is faster because of acceleration of hydrogen peroxide reaction with Fe^2+^ [19]. However, decolorization rate decreased at temperatures above 30°C after 30 min. This may be due to deactivation of several active sites of the enzyme because of denaturation of protein molecules by heating and therefore decreasing of *in-situ *hydrogen peroxide production rate.

### 3.5. pH Trend in the Reaction Media

**Figure 6:**
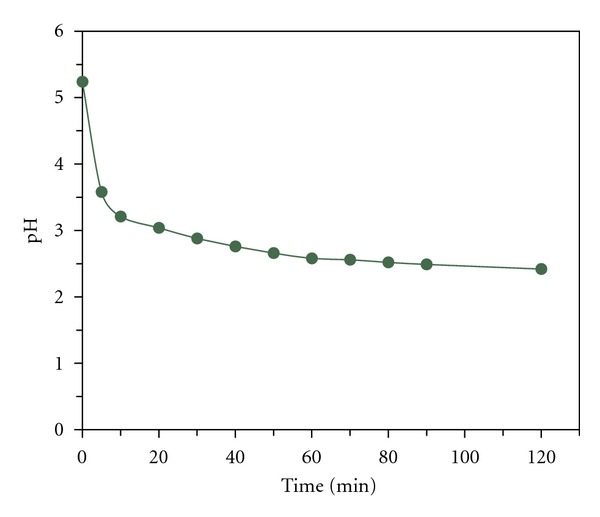
Variation of pH with time in experiments.

The variation of pH value was similar in all experiments and has been illustrated in Figure 6. The pH value in the reaction media was decreased from natural to about 3 which had synergic effect on Fenton process by stabilizing of Fe^2+^ ions. For determination of pH effect on MG concentration without bio-Fenton reaction, pHs of MG solutions with initial concentration of 15 mg/L were adjusted at 2.5, 3.5, 4.5, and 5.5 values. After 120 minutes decolorization of the samples was negligible.

## 4. Conclusion

In this study a new bio-Fenton process, involving enzymatic *in-situ* generation of H_2_O_2_, was developed for MG decolorization. Influence of several effective parameters on bio-Fenton process was investigated. It was found that bio-Fenton process was an efficient, simpler, and safe method for decolorization of MG. Decolorization of the dye was observed to be up to %78 during 120 min at the dye concentration of 20 mg/L without taking any control on pH.

## Figures and Tables

**Table 1:** Decolorization rate (mg/L·min) at different initial concentrations of MG at [Glucose]_0_ = 1 g/L, [enzyme]_0_ = 1000 U/L, [Fe^2+^]_0_ = 10 mM, and T=30°C.

Time (min)	Decolorization rate (mg/L·min)				
	at initial MG concentrations (mg/L) of				
	5	10	15	20	40
0–20	19.25	27.50	38.67	53.60	74.68
0–60	7.17	11.67	17.53	23.12	39.34
0–120	3.77	6.52	9.59	13.03	21.04
20–60	1.12	3.75	6.96	7.87	21.66

**Table 2:** Decolorization rate (mg/L·min) at different temperatures at [MG]_0_ = 15 mg/L, [Glucose]_0_ = 1 g/L, [enzyme]_0_ = 1000 U/L and [Fe^2+^]_0_ = 10 mM.

Time (min)	Decolorization rate (mg/L·min)			
	at temperatures (°C) of			
	15	30	35	40
0–20	30.51	38.68	37.50	40.035
0–60	17.65	17.53	17.37	17.48
0–120	9.55	9.59	8.90	8.36
